# Accurate Stride-Length Estimation Based on LT-StrideNet for Pedestrian Dead Reckoning Using a Shank-Mounted Sensor

**DOI:** 10.3390/mi14061170

**Published:** 2023-05-31

**Authors:** Yong Li, Guopei Zeng, Luping Wang, Ke Tan

**Affiliations:** 1 School of Biomedical Engineering, Sun Yat-sen University, Shenzhen 518107, China; liyong67@mail2.sysu.edu.cn; 2 School of Electronics and Communication Engineering, Sun Yat-sen University, Shenzhen 518107, China; zenggp6@mail2.sysu.edu.cn; 3 Educational Technology Center, The PLA General Hospital, Beijing 100853, China

**Keywords:** pedestrian dead reckoning, stride-length estimation, Transformer model, Kalman filter, inertial measurement unit (IMU)

## Abstract

Pedestrian dead reckoning (PDR) is a self-contained positioning technology and has been a significant research topic in recent years. Pedestrian-stride-length estimation is the core part of the PDR system and directly affects the performance of the PDR. The current stride-length-estimation method is difficult to adapt to changes in pedestrian walking speed, which leads to a rapid increase in the error of the PDR. In this paper, a new deep-learning model based on long short-term memory (LSTM) and Transformer, LT-StrideNet, is proposed to estimate pedestrian-stride length. Next, a shank-mounted PDR framework is built based on the proposed stride-length-estimation method. In the PDR framework, the detection of pedestrian stride is achieved by peak detection with a dynamic threshold. An extended Kalman filter (EKF) model is adopted to fuse the gyroscope, accelerometer, and magnetometer. The experimental results show that the proposed stride-length-estimation method can effectively adapt to changes in pedestrian walking speed, and our PDR framework has excellent positioning performance.

## 1. Introduction

Indoor-positioning technology has been widely applied in indoor navigation [1], disaster rescue [2], physical training, and health monitoring [3]. The rapid development of satellite navigation has greatly increased the application of positioning technology. However, due to the lack of satellite-navigation signals in indoor environments, accurate indoor positioning is still a challenging problem [4,5]. Pedestrian dead reckoning (PDR) based on IMU sensors, including gyroscopes, accelerometers, and magnetometers, has been a research focus in recent years. Compared with ultra wideband (UWB) [6], wireless fidelity (WIFI) [7,8], Bluetooth [9], and other positioning technologies, IMU-based PDR technology does not require the addition of any further infrastructure in indoor environments. It is a self-contained positioning method, which can reduce the costs of constructing and maintaining infrastructure, and has a wider range of applications. However, due to the large error of the inertial sensors in the micro-electro-mechanical system (MEMS) and the diversity of pedestrian walking modes, the positioning accuracy of current PDR methods is still unsatisfactory. Therefore, it is necessary to study new PDR methods to improve the performance of PDR.

Most of the previous studies focused on foot-mounted PDR assisted by zero velocity update (ZUPT). However, this kind of PDR system can only be used for pedestrian-position estimation, and it is difficult to extend to other fields, such as pedestrian-posture monitoring and activity recognition. The installation of low-cost MEMS sensors on the shanks of pedestrians can be used for pedestrian-trajectory estimation and human-activity recognition at the same time, which enable more comprehensive monitoring of pedestrians’ movement status. In other words, a PDR system combined with pedestrian-activity recognition is more valuable to users. Therefore, we developed the shank-mounted PDR technology based on our previous work on wearable human-activity-recognition technology [10]. As it is difficult to detect the zero-velocity interval, the step-and-heading system (SHS) is often used in the non-foot-mounted PDR. The positions of pedestrians are constantly updated based on the detected step, estimated stride length, and estimated heading in the SHS [11,12].

Stride-length estimation is an important part of the PDR system, and its error is the main source of the error in this system. Many previous studies used the acceleration-based nonlinear model [13] or the frequency-based linear model [14,15] to estimate pedestrian-stride length. The Weinberg model [16,17] is widely used, which constructs a nonlinear function based on the maximum and minimum accelerations to estimate stride length. Yao et al. [18] proposed a new model by combining the Weinberg model and the pedestrian walking frequency. To improve the accuracy of the model, the parameters were adjusted according to different walking patterns. Zhao et al. [13] adopted different stride-length-estimation models for three scenarios: walking on a plane, moving up and down stairs, and the transition step. Their model’s parameters need to be tuned according to the physical characteristics of pedestrians. These works report that their stride-length-estimation models have high accuracy; however, the main drawback of these works is that their models cannot adapt to changes in pedestrian walking speed and the differences between individuals. Deep neural networks have strong abilities in feature learning and data fitting, which have been widely used in image recognition [19], natural language processing [20], and other fields. In recent years, some scholars have attempted to introduce the deep-learning method into stride-length estimation. Gu et al. [21] used stacked autoencoders to learn the features of sensor data, and adopted a linear-regression layer to predict the pedestrian-stride length. Wang et al. [22] built a deep-learning model based on long short-term memory (LSTM) and an autoencoder to estimate stride length. Klein et al. [23] proposed a deep-learning model named StepNet to estimate the pedestrian-stride length. These stride-length-estimation methods based on deep learning show good results; however, it is still necessary to further study new stride-length-estimation methods to improve the accuracy of PDR systems.

Although significant work has been performed on PDR [13,24,25], the creation of an accurate PDR system still poses significant challenges due to various factors, specially changes in pedestrian walking speed. The different walking speeds and walking habits of pedestrians can create serious errors in some of the current methods of stride-length estimation, eventually leading to the failure of pedestrian-trajectory estimation. To improve the accuracy of PDR, in this article, we provide an improved PDR method using a shank-mounted IMU sensor. We propose an accurate stride-length-estimation method based on deep learning to adapt to the different movement speeds of pedestrians and apply it to the PDR framework. The contributions of this article are as follows:

(1) A new deep-learning model based on LSTM and Transformer, LT-StrideNet, is proposed to estimate pedestrian-stride length.
(2) A shank-mounted PDR framework is provided, which is a supplementary scheme to the foot-mounted PDR.
(3) Tests were completed using the our self-developed IMU module, including indoor and outdoor positioning.

The remainder of this paper is organized as follows. In Section 2, the method in this paper is described. In Section 3, the experimental setup and results are presented. In Section 4, the experimental results are discussed. In Section 5, the conclusion of this paper is given.

## 2. Materials and Methods

**Figure 1:**
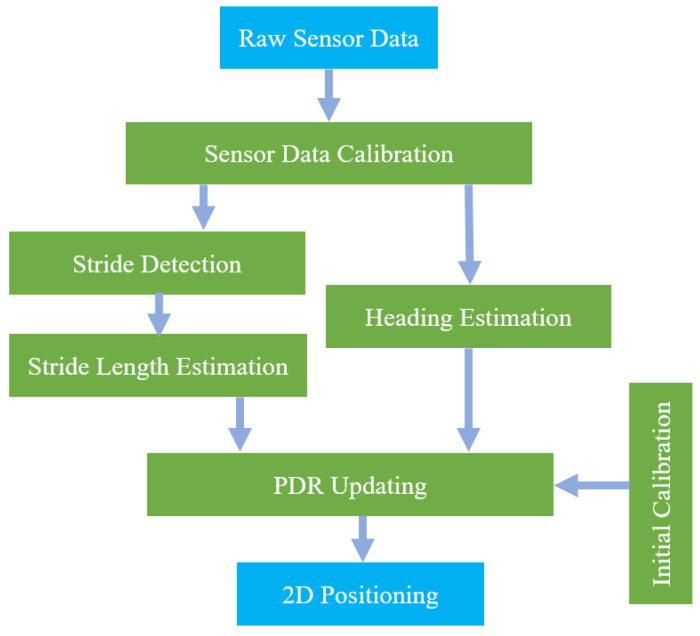
Flowchart of the PDR method.

A flowchart of the PDR method is shown in Figure 1, which consists of six parts: (1) sensor-data calibration, (2) stride detection, (3) stride-length estimation, (4) heading estimation, (5) initial calibration, and (6) updating of the PDR. The sensor-data calibration includes the calibration of sensor and filtering of sensor data. The stride-detection module detects pedestrian-stride events using acceleration or angular velocity. The pedestrian-stride length can be estimated by some methods, such as deep learning, linear regression, and empirical relationships. The heading estimation is realized by fusing the gyroscope, accelerometer, and magnetometer. The initial calibration includes the initial calibration of position and attitude. For the PDR system, the initial position is generally set to the origin. When the heading and stride length of pedestrians are obtained, the position of each stride can be calculated through the PDR motion model, as follows:

(1) $$X_{k}=X_{k-1}+L_{k}\left[\begin{array}{c} \cos(\varphi_{k})\\ \sin(\varphi_{k}) \end{array}\right]$$

where $X_{k}=(x_{k},y_{k})$ is the position of pedestrian on the 2D plane at k-th stride, $L_{k}$ is the stride length, and $\varphi_{k}$ is the heading.

According to the working process of PDR, the errors of pedestrian position accumulate with the increase in the number of strides. As a key part of PDR method, its error directly determines the error of pedestrian position. The classical Weinberg model calculates the stride length by the following Equation:

(2) $$L=k_{stride}\times \sqrt[{4}]{a^{\max}-a^{\min}}$$

where $a^{\max}$ and $a^{\min}$ are the maximum and minimum values of norm of acceleration. The $k_{stride}$ is the personalized constant coefficient, which needs to be tuned for each pedestrian. Due to the differences in leg length, weight, and stride frequency of pedestrians, the $k_{stride}$ is different for each pedestrian. On the other hand, even for the same pedestrian, the $k_{stride}$ changes continuously at different speeds. As a result, a pre-set $k_{stride}$ leads to a large estimation error. Stride-length estimation is a typical regression problem. The automatic learning ability of the deep neural network gives it excellent fitting properties. The LSTM is a popular recurrent neural network [26], which has been widely used in forms of time-series processing such as natural language processing, human-activity recognition, etc. It can effectively extract the dependencies between different time steps in time series. The Transformer model [27], which has emerged in recent years, has also received widespread attention in the field of computer vision. Transformer is a neural network based on self-attention mechanism. The self-attention mechanism can enhance the classification and fitting ability of model by automatically assigning greater weight to important and valuable features. Introducing Transformer into the LSTM network has the potential to further improve the accuracy of the model. Therefore, we designed a new stride-length-estimation model by combining LSTM and Transformer.

### 2.1. Stride-Length-Estimation Method Based on LSTM and Transformer

**Figure 2:**
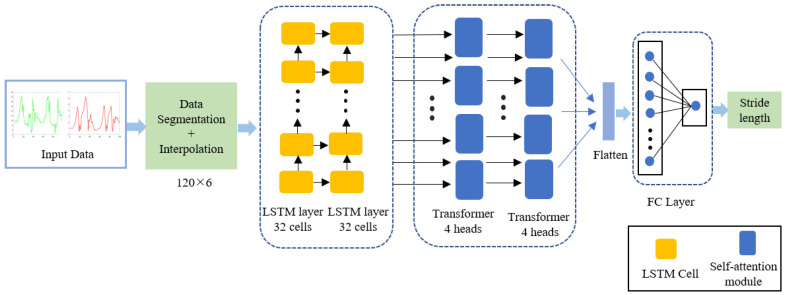
The architecture of the proposed stride-length-estimation model.

An end-to-end neural network, which we named LT-StrideNet, is proposed. The architecture of our model is shown in Figure 2. The measurements from the accelerometer and gyroscope are selected as the input data and need to be segmented and interpolated. Next, the pre-processed data are fed to the ST-StrideNet for stride-length estimation.

#### 2.1.1. Segmentation of Sensor Data

The sensor data can be divided into many segments, according to the beginning and end moments of each stride, by the stride-detection module. The measurements of accelerometer and gyroscope within a stride are expressed as follows:

(3) $$\left\{\begin{array}{l} a_{k}^{x}={\left[a_{t}^{x},a_{t+1}^{x},\cdot \cdot \cdot ,a_{t+m-1}^{x}\right]}^{T}\\ a_{k}^{y}={\left[a_{t}^{y},a_{t+1}^{y},\cdot \cdot \cdot ,a_{t+m-1}^{y}\right]}^{T}\\ a_{k}^{z}={\left[a_{t}^{z},a_{t+1}^{z},\cdot \cdot \cdot ,a_{t+m-1}^{z}\right]}^{T}\\ w_{k}^{x}={\left[w_{t}^{x},w_{t+1}^{x},\cdot \cdot \cdot ,w_{t+m-1}^{x}\right]}^{T}\\ w_{k}^{y}={\left[w_{t}^{y},w_{t+1}^{y},\cdot \cdot \cdot ,w_{t+m-1}^{y}\right]}^{T}\\ w_{k}^{z}={\left[w_{t}^{z},w_{t+1}^{z},\cdot \cdot \cdot ,w_{t+m-1}^{z}\right]}^{T} \end{array}\right.$$

where $\left[a_{k}^{x},a_{k}^{y},a_{k}^{z}\right]$ and $\left[w_{k}^{x},w_{k}^{y},w_{k}^{z}\right]$ are the outputs of the accelerometer and gyroscope respectively, k is the count of strides, t is the start time of the stride, and m is the length of samples during a stride. Due to the different duration of each stride, and since the neural network only receives fixed-size segments, the input data are expanded or compressed to the same size by spline interpolation. The input of neural network is expressed as follows:

(4) $$x_{input}=[a_{k}^{x},a_{k}^{y},a_{k}^{z},g_{k}^{x},g_{k}^{y},g_{k}^{z}]$$

which has dimensions of 120 × 6.

#### 2.1.2. The Deep-Learning Model for Stride-Length Estimation

First, the two-layer LSTM encoder extracts the temporal features of the sensor data. In a LSTM cell, $x_{t}$, $h_{t}$, and $c_{t}$ represent the input vector, hidden state, and long-term state. There are three gates in the LSTM cell, namely input, forget, and output. According to the theory of LSTM, the calculation of each component in the LSTM cell at current time step is as follows [22]:

(5) $$i_{t}=\sigma (U_{i}\cdot x_{t}+W_{i}\cdot h_{t-1}+b_{i})$$

(6) $$f_{t}=\sigma (U_{f}\cdot x_{t}+W_{f}\cdot h_{t-1}+b_{f})$$

(7) $$o_{t}=\sigma (U_{o}\cdot x_{t}+W_{o}\cdot h_{t-1}+b_{o})$$

(8) $${\tilde{c}}_{t}=\tanh(U_{c}\cdot x_{t}+W_{c}\cdot h_{t-1}+b_{c})$$

(9) $$c_{t}=f_{t}⊙c_{t-1}+i_{t}⊙{\tilde{c}}_{t}$$

(10) $$h_{t}=o_{t}⊙\tan h(c_{t})$$

where $h_{t-1}$ is the hidden state of last LSTM cell, $\left[U_{i},W_{i}\right]$, $\left[U_{f},W_{f}\right]$, and $\left[U_{o},W_{o}\right]$ are the weight matrices of the input gate, forgetting gate, and output gate, respectively. The $\left[b_{i},b_{f},b_{o}\right]$ is the corresponding bias. The σ is the sigmoid function, and ⊙ denotes element multiplication. With the special mechanism of LSTM, the long-term dependence of time series can be extracted.

**Figure 3:**
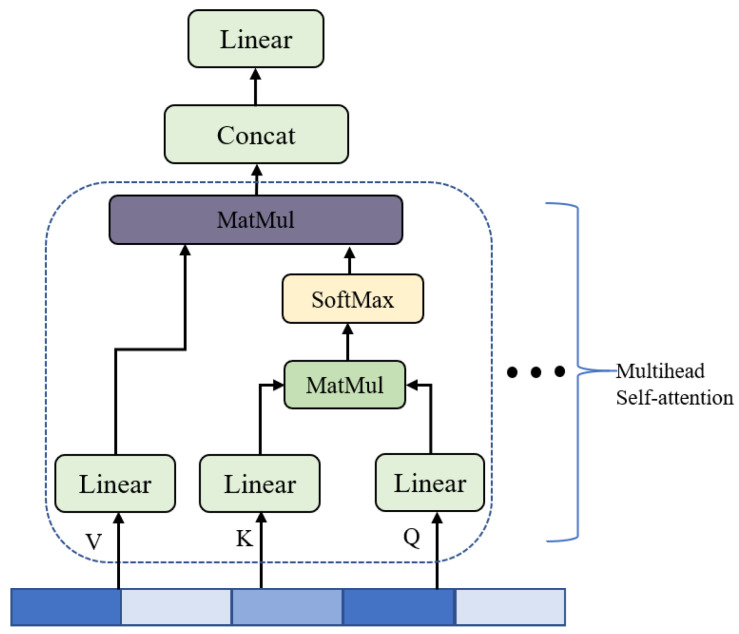
The overview of the multi-head self-attention.

In our model, we add the two-layer Transformer encoder after the LSTM layer and set four headers per layer. Multi-head self-attention (MHA) is the core of Transformer architecture, the aim of which is to enable the model to focus on the important time steps in feature sequences. The MHA consists of multiple self-attention operations that are executed in parallel. Firstly, the self-attention mechanism determines the relative weight of each time step in a sequence by computing its similarity to other time steps within the sequence. Subsequently, the representation of each time step is transformed using these relative weights. The overview of MHA [27] is shown in Figure 3. In the self-attention, three identical sequences $Q\in {\mathbb{R}}^{t\times d}$, $K\in {\mathbb{R}}^{t\times d}$, and $V\in {\mathbb{R}}^{t\times d}$, are referred to as query, key, and value, respectively. First, they are projected to the same dimensions by three learned linear transformations,

(11) $$Q_{h}=QW_{h}^{Q}\in {\mathbb{R}}^{t\times d^{\prime }}$$

(12) $$K_{h}=KW_{h}^{K}\in {\mathbb{R}}^{t\times d^{\prime }}$$

(13) $$V_{h}=VW_{h}^{V}\in {\mathbb{R}}^{t\times d^{\prime }}$$

where $d^{\prime }=\frac{d}{n_{h}}$ and $n_{h}$ are the number of heads, and $W_{h}^{Q}$, $W_{h}^{K}$, and $W_{h}^{V}$ are the projection matrices to be learned. The dot product is implemented between the query considered as the transformed matrix of a specific time step and the key matrix of every other time step. Next, the softmax is applied on the scaled result of dop product to obtain the attention scores. Finally, the attention scores are used to obtain the weighted representation of the value matrix V:

(14) $$h_{i}^{sa}(Q,K,V)=\mathrm{softmax}(\frac{QK^{T}}{\sqrt{d}})V$$

In the MHA, the self-attention is computed in parallel (multi-head) to capture the correlation information of input sequences from different representation subspaces. That is, the operations within the dotted box in Figure 3 are executed multiple times in parallel. The outputs of all the heads are concatenated across the channel dimension and once again projected to the same dimension of input sequences using the learned matrix $W_{0}$, as follows,

(15) $$M_{ha}=W_{0}\cdot Concat(h_{1}^{sa},h_{2}^{sa},h_{3}^{sa},\ldots ,h_{h_{n}}^{sa}).$$

By performing the above process, the MHA can effectively focus on the important part of the feature sequence. To obtain the pedestrian-stride length, the outputs of Transformer encoder are mapped to the specified size by a fully connected layer. The Dropout technique is used in the Transformer encoder and fully connected layer to prevent overfitting of the model.

#### 2.1.3. The Training of Stride-Length-Estimation Model

When the model is trained, the overall objective is to minimize the following error loss function:

(16) $$J=\frac{1}{N}\sum_{i=1}^{N}{\left(y_{i}-{\hat{y}}_{i}\right)}^{2}$$

where $y_{i}$ is the actual stride length and ${\hat{y}}_{i}$ is the output of the fully connected layer. The stochastic gradient descent algorithm is used to search for the optimal model parameters. Once the training of the model is complete, the model can be used to estimate pedestrian-stride length. The complete procedure of our stride-length-estimation method is shown in Algorithm 1.
**Algorithm 1:** The proposed stride-length-estimation method**Input:** training data with actual stride length, test data without actual stride length **Output:** the estimated stride length 1//Data preprocessing 2 **for** each stride 3        Split the sensor data according to the stride event 4        Extract the sensor data and actual length of each stride 5        Expand or compress the sensor data to a fixed size 6 **end for**
7//Model training 8 Build and train the stacked LSTM–Transformer model shown in Figure 2 using the training dataset 9 Save the trained model 10//Model test 11 Predict the pedestrian-stride length using the saved model

### 2.2. The PDR Framework with Proposed Stride-Length-Estimation Method

**Figure 4:**
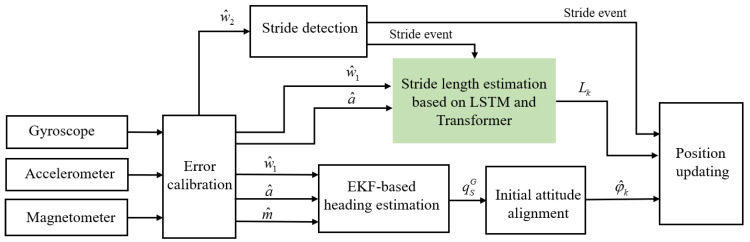
Overall architecture of the proposed PDR framework. ${\hat{w}}_{1}$: the angular rate with bias removed, ${\hat{w}}_{2}$: the filtered angular rate, $\hat{a}$: the calibrated acceleration, $\hat{m}$: the calibrated output of magnetometer, $q_{S}^{G}$: the quaternion representing the sensor attitude, ${\hat{\varphi }}_{k}$: the heading of pedestrian at k-th stride, $L_{k}$: the stride length of pedestrian at k-th stride.

Based on the above stride-length estimation method, we built a PDR framework, as shown in Figure 4. Our PDR framework adopts a low-cost 9-aixs sensor to provide raw sensor data. In this paper, the output of gyroscope is preprocessed twice. First, the fixed bias is removed from the output of gyroscope to obtain the ${\hat{w}}_{1}$. Next, a moving-average filter is used on ${\hat{w}}_{1}$ to obtain ${\hat{w}}_{2}$. The ${\hat{w}}_{1}$ is used for stride-length estimation and heading estimation, while the ${\hat{w}}_{2}$ is used for stride detection. The magnetometer is fused with the gyroscope and accelerometer by the extended Kalman filter (EKF) model. The purpose of the initial attitude alignment of IMU is to determine the initial attitude error of pedestrians in the navigation coordinate system (NCS).

#### 2.2.1. Sensor-Error Calibration

In the static state, the output of an ideal accelerometer should be the local gravitational acceleration. The output vectors of the accelerometer at different attitudes should be distributed over a sphere with radius g. Due to manufacturing techniques, the output of an accelerometer at different attitudes may be an ellipsoid. The accelerometer is calibrated by an ellipsoidal fitting algorithm [28].

Due to soft-iron, hard-iron, and scale-factor errors, the output of the magnetometer is an ellipsoid that deviates from the origin. Similar to accelerometer calibration, the ellipsoidal fitting algorithm is also used to calibrate the magnetometer. In this article, we mainly consider weakening the hard-iron and scale -actor errors. Before the experiment, we kept the magnetometer away from the soft-iron material and continuously rotated the magnetometer along different coordinate axes for a few minutes to collect the local magnetic field. The output of magnetometer was then substituted into the ellipsoidal model and the model parameters were obtained by the ellipsoidal fitting algorithm. We used the offset vector Δm and the scale transformation matrix $C_{sf}$ to calibrate the raw measurement of magnetometer m, as follows:

(17) $$\hat{m}=C_{sf}(m+\Delta m).$$

**Figure 5:**
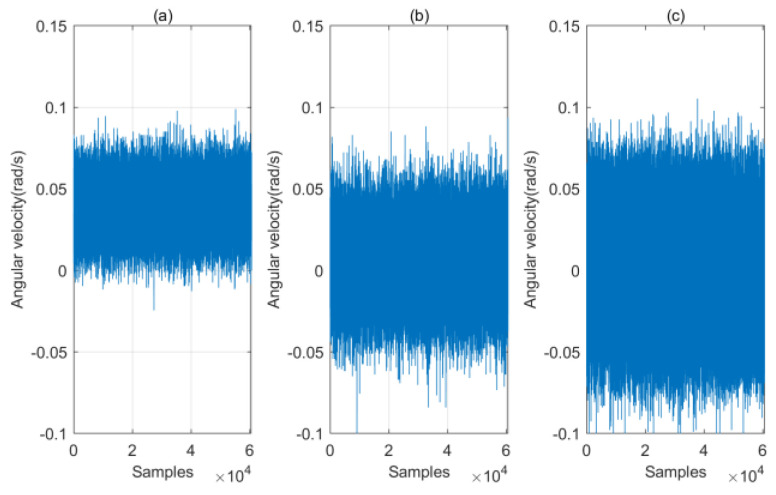
The output of gyroscope in the static state: (**a**) axis *x*, (**b**) axis *y*, (**c**) axis *z*.

To obtain the bias of the gyroscope, the output of the gyroscope in the static state for more than 20 min was collected, as shown in Figure 5. Next, the average value of each axis was calculated and used as the bias of gyroscope. The output of gyroscope subtracts the bias $w_{B}=[0.039,0.0058,0.0012]$ before it is fed into the PDR algorithm.

#### 2.2.2. Stride-Detection Method

Traditional pedestrian-stride detection is performed by setting a fixed threshold to detect the peak of acceleration waveform [29]. This method has an obvious shortcoming. When the speed of pedestrian changes obviously, the fixed threshold leads to the detection of a false peak, resulting in error in stride detection. To search the local maximum peak, we introduce two threshold-based constraints. That is, the constraint of peak amplitude and the constraint of the time interval between two consecutive peaks. For the constraint of peak amplitude, a dynamic threshold was designed to adapt to changes in pedestrian speed. We chose the norm of angular rate as the input of stride-detection module, which is represented as:

(18) $$g_{i}=\sqrt{w_{ix}^{2}+w_{iy}^{2}+w_{iz}^{2}}$$

Before detecting the peaks, moving-average filtering on the norm of angular rate was performed to eliminate the high-frequency noise. A sliding window with length of three was used to judge the peak and the moment when the peak appeared was recorded:

(19) $$if(g_{i-1}\geq g_{i-2}{\&\&\;g}_{i-1}\geq g_{i}{\&\&g}_{i-1}\geq th_{-}w)\;\;\;pt_{k}=i-1$$

where $th_{-}w$ is a threshold obtained according to the amplitude of the wave peak. By calculating the difference between the maximum and minimum values of the input data within the last second, we can obtain the dynamic threshold as follows

(20) $$th_{-}w=k_{stride}*(\max(g_{-}s(1,1:f))-\min(g_{-}s(1,1:f)))+C_{0}$$

where $k_{stride}>0$ is a coefficient set by experience, f is the sampling frequency of gyroscope, $g_{-}s(1,1:f)$ is the input data within the last second, $C_{0}>0$ is a constant to prevent detection of the small peaks. Next, the time difference between the current candidate peak and the previous candidate peak can be obtained. We assume that the true peak should ensure that the time difference is greater than a threshold. Therefore, the true peak can be judged as follows:

(21) $$if(dt=(tp_{k}-tp_{k-1})>th_{-}t)\;is_{-}stride=true$$

where $th_{-}t$ is another threshold obtained by tuning. Since the stride frequency of most pedestrians is between 0.5 and 2 Hz, $th_{-}t$ can be set to 0.5 s. At this point, it is considered that a pedestrian stride has been detected.

#### 2.2.3. EKF-Based Heading Estimation

Compared to Euler angle, the quaternion has no problem of angle singularity, and is widely used to represent the rotation of vectors. We use the quaternion as the state variable of the EKF model, which can be expressed as follows:

(22) $$x_{k}={\left[q_{0},q_{1},q_{2},q_{3}\right]}^{T}$$

where $q_{0}$ is the scalar part and $\left[q_{1},q_{2},q_{3}\right]$ is the vector part.

According to the quaternion kinematics of the gyroscope [30], the process model of EKF can be expressed as

(23) $$x_{k+1}=(I+\frac{T}{2}\Omega_{k}^{b})x_{k}+n_{k}=A_{k}x_{k}+n_{k}$$

where I is a 4×4 identity matrix, T is the sampling interval of sensor, and $n_{k}$ is the process noise with covariance matrix $Q=\sigma_{q}^{2}I$. The $\Omega_{k}^{b}$ is a skew symmetric matrix, expressed as follows:

(24) $$\Omega_{k}^{b}=\left[\begin{array}{cccc} 0 & -{\hat{\omega }}_{k}^{x} & -{\hat{\omega }}_{k}^{y} & -{\hat{\omega }}_{k}^{z}\\ {\hat{\omega }}_{k}^{x} & 0 & {\hat{\omega }}_{k}^{z} & {\hat{\omega }}_{k}^{y}\\ {\hat{\omega }}_{k}^{y} & -{\hat{\omega }}_{k}^{z} & 0 & {\hat{\omega }}_{k}^{x}\\ {\hat{\omega }}_{k}^{z} & {\hat{\omega }}_{k}^{y} & -{\hat{\omega }}_{k}^{x} & 0 \end{array}\right]$$

where ${\hat{w}}_{k}=w_{k}-w_{B}$ is the angular velocity and $w_{B}=\left[w_{B}^{x},w_{B}^{y},w_{B}^{z}\right]$ is the bias of the gyroscope. The measurement model of EKF can be expressed as

(25) $${\left[z_{k}^{a},z_{k}^{m}\right]}^{T}=\left[\begin{array}{c} C_{n}^{b}(q_{k})r_{ak}+v_{ak}\\ C_{n}^{b}(q_{k})r_{mk}+v_{mk} \end{array}\right]=\left[\begin{array}{c} {\hat{z}}_{a}^{k}\\ {\hat{z}}_{k}^{m} \end{array}\right]+\left[\begin{array}{c} v_{k}^{a}\\ v_{k}^{m} \end{array}\right]$$

where $z_{k}^{a}={\left[a_{x},a_{y},a_{z}\right]}^{T}$, $z_{k}^{m}={\left[m_{x},m_{y},m_{z}\right]}^{T}$ are the measurements of accelerometer and magnetometer, respectively. The $C_{n}^{b}(q_{k})$ is the rotation matrix. The $r_{ak}={\left[0,0,g\right]}^{T}$ is the gravity vector. The $r_{mk}=[b_{x}^{n},0,b_{z}^{n}]$ is the Earth’s magnetic field and $r_{mk}$ can be written as

(26) $$r_{mk}=\left[\sqrt{h_{x}^{2}+h_{y}^{2}},0,h_{z}\right]$$

where the magnetic field $h^{m}=(h_{x},h_{y},h_{z})$ in the NCS (north, east, sky) can be computed by $h^{m}(q_{k})=C_{n}^{b}{}^{T}(q_{k})z_{k}^{m}$. The $v_{k}=[v_{k}^{a};v_{k}^{m}]$ is the measurement noise with covariance matrix $R=\left[\begin{array}{cc} \sigma_{a}^{2}\cdot I_{3\times 3} & 0_{3\times 3}\\ 0_{3\times 3} & \sigma_{m}^{2}\cdot I_{3\times 3} \end{array}\right]$. The Jacobian matrix of the measurement model is $H_{k}=\left[\begin{array}{cc} \frac{\partial {\hat{z}}_{a}^{k}}{\partial x_{k}}; & \frac{\partial {\hat{z}}_{k}^{m}}{\partial x_{k}} \end{array}\right]$. According to [12], the heading of sensor can be calculated by

(27) $$\varphi =\arctan\left[\frac{2(q_{1}q_{2}+q_{0}q_{3})}{q_{0}^{2}+q_{1}^{2}-q_{2}^{2}-q_{3}^{2}}\right].$$

When the pedestrians start walking, the ferromagnetic interference contaminates measurements of magnetometer, especially in indoor environments. We propose a threshold-based strategy to suppress this ferromagnetic disturbance. To use the reliable output of magnetometer, the following judgment is made at each sampling moment:

(28) $$if(\left|\left\|{\hat{m}}_{k}\right\|-m_{earth}\right|<Th_{mag})$$

where $\left\|{\hat{m}}_{k}\right\|$ is the norm of magnetometer measurement ${\hat{m}}_{k}$ and $m_{earth}$ is the norm of Earth’s magnetic field. The $Th_{mag}$ is a threshold preset by the experimenter and we set it to 10 uT. If the above formula is true, it is considered that the measurement of magnetometer is reliable. Measurement of magnetometer is used to estimate pedestrian heading through EKF. Otherwise, magnetometer measurement is discarded.

#### 2.2.4. The Initial Attitude Alignment

The initial attitude alignment is a necessary part of wearable motion tracking [31]. It is known that

(29) $$q_{B}^{G}=q_{S}^{G}⊗q_{B}^{S}$$

The goal of initial attitude alignment is to obtain the rotation quaternion $q_{B}^{S}$ from the body-coordinate system (BCS) to the sensor-coordinate system (SCS). As the accelerometer only senses the Earth’s gravity at static status, the initial sensor attitude represented by Euler angle can be calculated by the output of the accelerometer and magnetometer, which are as follows:

(30) $$\theta_{init}^{pitch}=-\arcsin(a_{x}/g)$$

(31) $$\phi_{init}^{roll}=\mathrm{atan}2(a_{y},a_{z})$$

(32) $$\varphi_{init}^{yaw}=-\mathrm{atan}2(m_{y}c\phi -m_{z}s\phi ,m_{x}c\theta +m_{y}s\phi s\theta +m_{z}c\phi s\theta )$$

The c and s are the abbreviations of cos and sin, respectively, in (32). Therefore, the initial quaternion $q_{S}^{G}{}_{init}$ can be computed by the Euler angle to quaternion. When a pedestrian stands upright and their hand is straight in the horizontal plane, this posture is called the T-pose [31]. In the T-pose, the BCS of pedestrian coincides with the NCS when they face the *x*-axis of NCS. Therefore, there is $q_{S}^{G}=q_{S}^{B}$ in that posture. As $q_{B}^{S}=q_{S}^{B}{}^{*}$, the pedestrian only needs to be in the T-pose and face the north to obtain $q_{S}^{B}$. Next, the initial attitude calibration is completed by Equation (29).

## 3. Experiments and Results

### 3.1. Introduction to the IMU Module

**Figure 6:**
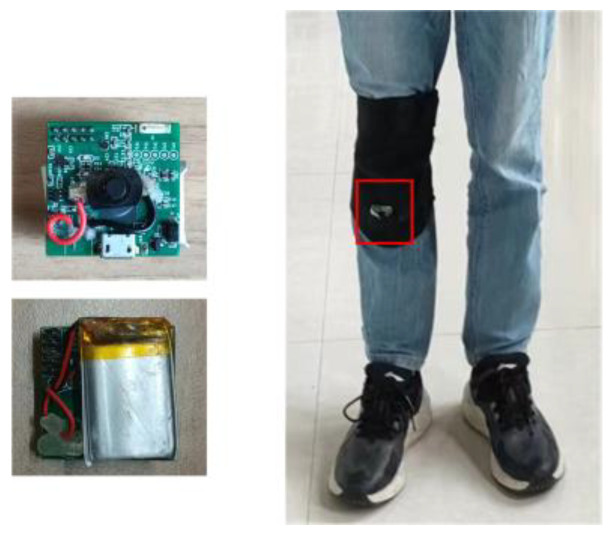
The IMU module and wearing position of the IMU.

In the experiment, a self-developed IMU module was used to collect the inertial data. We attached the IMU to the shanks of volunteers, just below the knee. All the volunteers were students from our research group. The IMU module and the wearing position of the IMU are shown in the Figure 6. The IMU contains a nine-axis MEMS sensor (MPU9250), which includes a three-axis gyroscope, a three-axis accelerometer, and a three-axis magnetometer. The circuit board is powered by a lithium battery. A microprocessor (TI CC2642) with Bluetooth 5.0 is integrated into the circuit as a control and communication unit. The sensor data are first stored on a flash chip, and are then transmitted to the computer through the serial port for processing. The collection of the raw sensor data is controlled by a smartphone and the control commands (starting collection, ending collection) are sent to the IMU by Bluetooth. Some of the sensor’s parameters are shown in Table 1.

For the EKF model, we rely on the empirical tuning of the parameters to obtain the variance matrix of the process noise and measurement noise. By minimizing the error between the estimated trajectory and the reference path, the parameters are finally tuned and determined, which is expressed as follows:

(33) $$Q=0.00001\times I_{4\times 1}$$

(34) $$R=\left[\begin{array}{cc} 0.018\times I_{3\times 3} & 0_{3\times 3}\\ 0_{3\times 3} & 0.337\times I_{3\times 3} \end{array}\right]$$

where Q is the covariance of the process noise, $0.018\times I_{3\times 3}$ is the covariance of the accelerometer noise, and $0.337\times I_{3\times 3}$ is the covariance of the magnetometer noise.

### 3.2. Training of Our Stride-Length-Estimation Model

**Figure 7:**
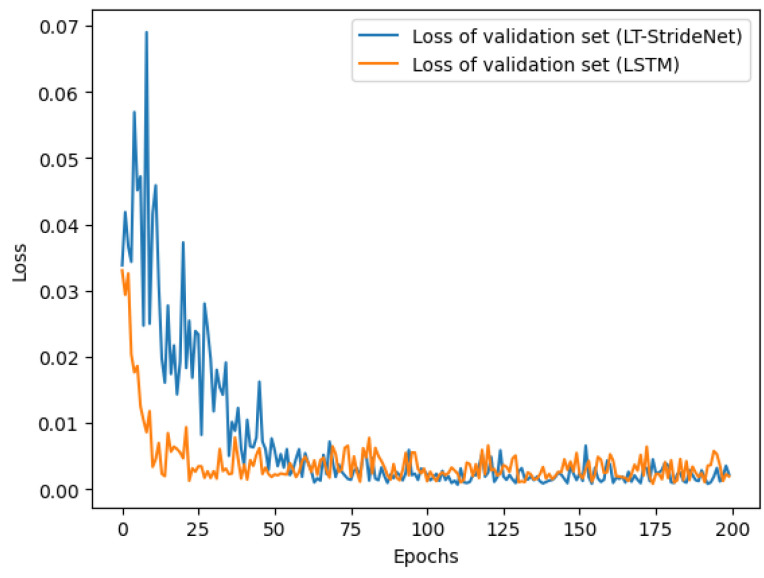
The loss of training set and validation set.

To create the dataset for training the stride-estimation model, four male volunteers wore the IMU sensor to collect inertial data in three motion modes (slow walking, normal walking, and fast walking). The physical characteristics of the volunteers are shown in Table 2, and volunteers 1 and 2 participated in the subsequent positioning experiment. The personalized coefficient in the Weinberg model is also listed. The volunteers were asked to walk at a uniform speed along three straight paths with lengths of 40 m, 73.2 m, and 94.4 m, and to record the actual number of strides taken during each walk. We assumed that the length of each stride was equal when the pedestrian walked at a uniform speed. Therefore, the actual stride length in each trajectory was obtained from the number of strides and the total length. The resulting stride lengths were added to the dataset as labels. The speeds of the volunteers ranged from 3.5 km/h to 7 km/h. Finally, we created a dataset with 1843 data segments and used 80% of the dataset for training and the remaining for validation. The details of the dataset are presented in Table 3. Our proposed model was implemented through PyTorch in Windows 10. The hyperparameters of our stride-estimation model are listed in Table 4, which were obtained through tuning. The loss of the training set and validation set during the process of the model training is shown in Figure 7. After training for 100 epochs, the loss remained stable. The mean validation losses of our model for the last 20 epochs was 0.0018 ± 0.0008, while the mean validation losses of the two-layer LSTM model were 0.0028 ± 0.0012. The results of the three iterations of the training show that our model had a 30.1% reduction in average validation loss compared to the LSTM model. These results illustrate the effectiveness of Transformer in improving the model’s performance.

### 3.3. Collection of Experimental Data for Pedestrian Positioning

Volunteers 1 and 2 participated in the data collection for the positioning experiment. We first tested the performance of the stride-detection and stride-length-estimation modules in three motion modes. The volunteers were asked to walk along paths with a known length of 94.4 m and the total numbers of strides they took were recorded.

**Figure 8:**
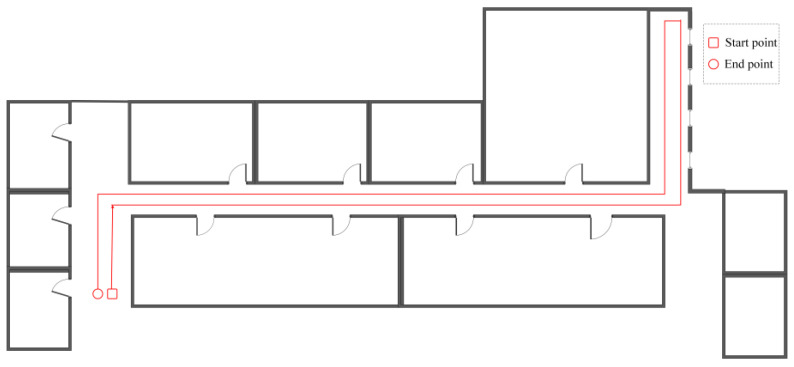
Plan of the indoor scene and reference path.

Next, we selected a path with turns in the indoor experimental environment of a school building to test the positioning performance of the PDR method. The volunteers were asked to walk along the path with a normal gait. The walking test was repeated three times for every volunteer. Figure 8 is the plan of the indoor scene and the predefined reference path with a length of 155.2 m. We also conducted data collection in a 400-m outdoor playground.

We used the stride-counting error, end-to-end error, and distance error, which are defined as (35), (36), and (37), respectively, to evaluate the algorithm’s performance. The true coordinate of the end-position and the estimated coordinate of end-position are denoted as $\left(P_{tcx},P_{tcy}\right)$ and $\left(P_{ecx},P_{ecy}\right)$, respectively. First, we calibrated the initial attitude of the sensor, and then the true initial heading was determined by the average value of the heading-estimation results before the walking took place. For each specified path, the true heading of each straight line was derived. Before the experiment, we measured the side length of the rectangular path with a tape measure. Using the derived heading and side length of the rectangular, the reference path in the 2D coordinate system was finally obtained.

(35) $$e_{s}=\frac{\left|N_{es}-N_{as}\right|}{N_{as}}\times 100\%$$

(36) $$e_{e}=\frac{\sqrt{{\left(P_{ex}-P_{sx}\right)}^{2}+{\left(P_{ey}-P_{sy}\right)}^{2}}}{d_{ref}}\times 100\%$$

(37) $$e_{d}=\frac{abs(d_{measured}-d_{ref})}{d_{ref}}\times 100\%$$

where $N_{es}$ is the number of estimated strides and $N_{rs}$ is the number of actual strides. The $d_{measured}$ is the sum of all the estimated stride lengths and $d_{ref}$ is the length of the reference path.

### 3.4. Experimental Results

#### 3.4.1. Results of Stride Detection and Stride-Length Estimation

**Figure 9:**
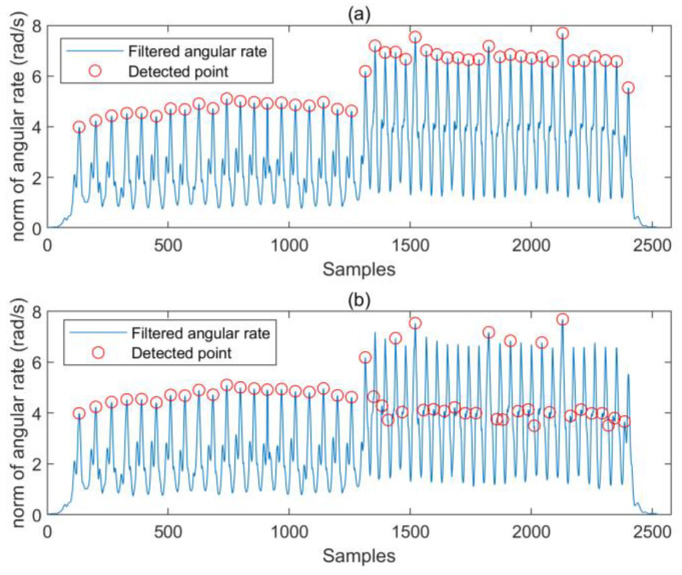
The peak-detection results of one trial; (**a**) the results of our method, (**b**) the results of the peak-detection method using a fixed threshold.

The results of the stride detection for one trial are shown in Figure 9. The volunteer was asked to walk at a normal speed for a period of time, and then to walk quickly. Our method accurately detected the true peaks, and the number of strides given by our method was consistent with the actual number of strides. When the speed of the pedestrian increased, false detection occurred using the method of peak detection with fixed thresholds ($th_{-}w=3.5$).

**Figure 10:**
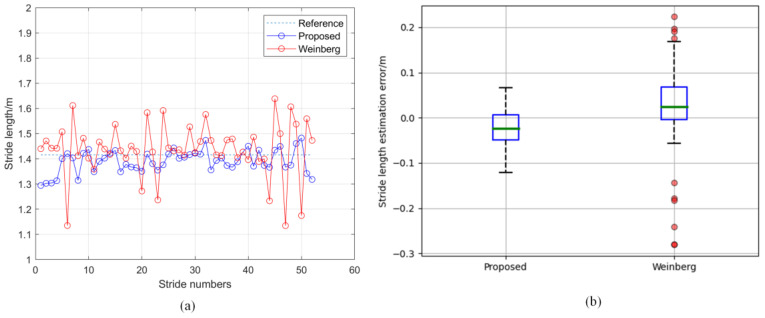
Results of stride-length estimation for one trial: (**a**) stride-length-estimation results of our method and Weinberg model, (**b**) estimation errors of the two methods.

Figure 10 shows the results of the step-length estimation for volunteer 1 when walking at a uniform speed. The stride lengths estimated by the two methods are shown in Figure 10a, and the error distributions of the two methods are shown in Figure 10b. It can be seen from the figure that the stride length obtained by our method had a smaller error than that obtained using the Weinberg model. Further, the experimental results of the walking tests at different walking speeds are shown in Table 5. It can be observed that the mean stride-counting error and mean distance error of the proposed method were (0.5 ± 0.9)% and (2.6 ± 1.3)%, respectively, while the mean distance error obtained by the PDR with the Weinberg model was (7.5 ± 3.9)%.

#### 3.4.2. Results of Indoor Positioning

**Figure 11:**
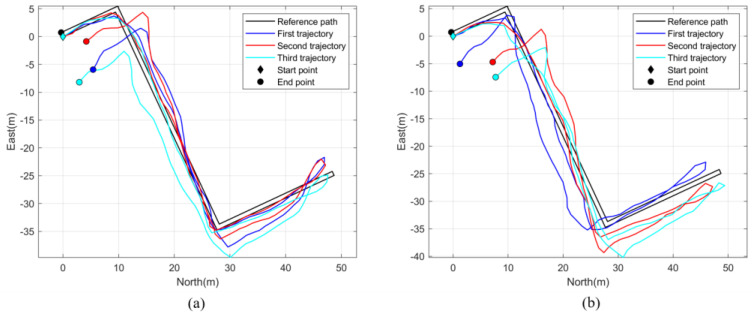
The estimated trajectories of two volunteers: (**a**) the trajectory of volunteer 1, (**b**) the trajectory of volunteer 2.

Figure 11 shows the estimated trajectories of two volunteers in the indoor scenes, where (a) is the trajectory of volunteer 1 and (b) is the trajectory of volunteer 2. Table 6 lists the results of the two volunteers. It can be seen from the table that the mean end-to-end error of our PDR method was 5.4 ± 1.6% and that the mean distance error was 3.0 ± 0.9%. Almost all of the volunteers’ strides were correctly detected.

#### 3.4.3. Results of Outdoor Positioning

**Figure 12:**
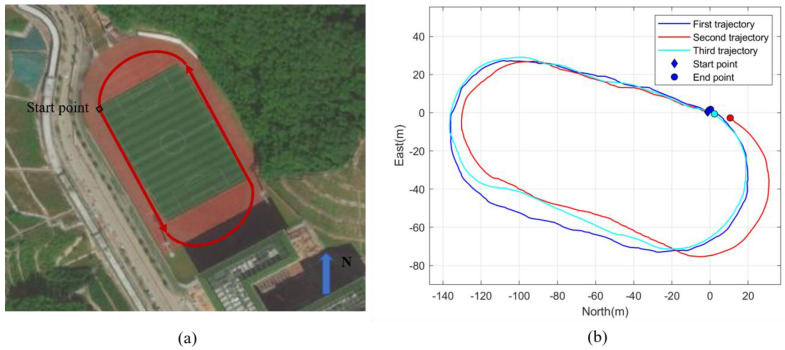
The estimated trajectory of pedestrian in the playground: (**a**) the actual path, (**b**) the estimated trajectory.

Figure 12 shows the estimated trajectory of volunteer 1 walking in the outdoor playground. The picture in Figure 12a is from Baidu Map. Three repeated tests were conducted at different times. Table 7 shows the results of the tests. It can be seen from the table that the end-to-end error of this outdoor test was 1.2 ± 1.3% and that the distance error was 1.8 ± 1.5%.

## 4. Discussion

### 4.1. Evaluation of Experimental Results

In order to improve the accuracy of the PDR, we proposed a stride-estimation method based on a deep-learning model, and built a shank-mounted PDR framework using the SHS. Our stride-detection method exhibited good performance in terms of the pedestrians’ walking speeds. As shown in Figure 9, the dynamic threshold is beneficial for responding to changes in waveform amplitude. In addition, constraints on the durations of strides can reduce the occurrence of false positives. As shown in Table 5, the distance error of our PDR method is significantly smaller than that of the PDR method with the Weinberg model. The compared PDR method differs from ours only in terms of the stride-length estimation. Therefore, our stride-length-estimation method has a significant advantage over the Weinberg model. The estimation error of the Weinberg model increases with the decrease in the pedestrian’s speed, while our method can adapt well to changes in pedestrian walking speed. Since it is data-driven, we expect that the performance of our method will be improved further.

According to Table 6, the mean end-to-end error of our PDR algorithm was 5.4 ± 1.6% in the indoor environment. As our test environment was an experimental building, the heading-estimation algorithm was susceptible to ferromagnetic interferences. Although we rejected some serious ferromagnetic interferences though the threshold, ferromagnetic interferences can still cause errors in pedestrian-heading estimation. It can be seen in Figure 11 and Table 7 that the experimental results of our PDR algorithm were better than those in the indoor environment. The mean end-to-end error and distance error were both below 2%. These results illustrate the excellent performance of our method in an environment with low magnetic interferences.

There are still several limitations to discuss. First, the algorithm needs to be tested in more environments and with more volunteers. In other words, the generalization of the algorithm needs further study. Second, some parameters in the algorithm need to be tuned manually. For example, the parameters in the EKF model need to be tuned according to the environment and sensor characteristics. The algorithms for automatically setting the parameters are not studied in this paper. Third, the ferromagnetic interference that occurs in indoor scenarios lead to the degradation of the algorithm’s performance. It is necessary to further study the heading-estimation algorithm against ferromagnetic disturbance.

### 4.2. Comparison with Related Works

We list some recent works on PDR based on wearable inertial sensors in Table 8. All the listed indicators were obtained from pedestrians walking on a flat surface. The number of participants is also listed in the table. In Hou and Bergmann’s work [24], the inertial sensor was placed on the head of a pedestrian. Their work had an EE of 1.3% and a DE of 2.1%. It should be mentioned that their experiments were conducted in an outdoor environment. Their results were close to those of our test in the outdoor environment. Zhao et al.’s work [13] exhibited a lower estimation error. In their work, the wearable sensor was attached to the ankle of the volunteer. Their tests were also conducted outdoors, and there were multiple personalized parameters in their stride-length-estimation method. The DE in Hajati et al.’s work [17] reached 0.45%; however, they did not provide the EE. They used the Weinberg model to estimate the stride length, and the accuracy of their method decreased as the walking speed changed.

Although the positioning error in Bai et al.’s work [32] was very small, they used a ZUPT-assisted PDR method. Its limitation is that the IMU sensor needs to be tied to the foot, making it difficult to achieve a more comprehensive monitoring of human activity. Our work uses a low-cost IMU sensor mounted on the shank of the pedestrian, which provides a feasible shank-mounted PDR scheme. In addition, the system proposed in this paper has the advantages of low cost, small size, and light weight. The proposed method can be easily extended to other applications, such as lower-limb postoperative-rehabilitation systems and physical training.

## 5. Conclusions

This paper proposed a new deep-learning model based on LSTM and Transformer to estimate pedestrian-stride length. To this end, a PDR framework was built using a shank-mounted sensor, assisted by a peak-based stride-detection method and an EKF-based heading-estimation model. The experimental results showed that our stride-length-estimation method can effectively adapt to changes in pedestrian walking speed, and that its error is significantly lower than that of the traditional Weinberg model. The experimental results on positioning showed that the end-to-end and distance errors of the proposed PDR method were 5.4% and 3.0% in the indoor environment. In the outdoor environment, the end-to-end and distance errors of the proposed PDR method were 1.2% and 1.8%, respectively. The results in this paper show that our PDR method has excellent positioning performance and promising application potential. The main limitation of our work is that the generalization of our method needs further investigation. Next, we will develop more robust positioning algorithms.

## Figures and Tables

**Table 1:** Sensor parameters.

Sensor	Accelerometer	Gyroscope	Magnetometer
Dimensions	3 axes	3 axes	3 axes
Dynamic range	±8 g	±2000 dps/s	±4800 uT
Bandwidth (Hz)	50	50	10
Nonlinearity (%)	±0.5	±0.1	±0.5
Noise density	400 $\mu g/\sqrt{\mathrm{Hz}}$	0.01 °/s/$\sqrt{\mathrm{Hz}}$	-
Bias stability	160 μg	74 °/h	-

**Table 2:** The physical characteristics of four male volunteers.

Volunteers	Height (m)	Age	Weight (kg)	$k_{length}$
1	1.78	33	67	0.77
2	1.75	25	75	0.82
3	1.72	25	60	-
4	1.68	24	65	-

**Table 3:** The details of our dataset for stride-length estimation.

Parameters	Value
Number of trajectories	52
Number of total data segments	1843
Number of data segments for walking slowly	718
Number of data segments for walking normally	601
Number of data segments for walking quickly	524

**Table 4:** The hyperparameters of our stride-length-estimation model.

Hyperparameters	Value
Number of LSTM cells per hidden layer	32
Number of LSTM layers	2
Number of Transformer-encoder layers	2
Number of heads per Transformer-encoder layer	4
Dropout	0.3
Loss function	MSE
Learning rate	0.001
Batch size	32
Optimizer	Adam
Epochs	200

**Table 5:** The results of the walking tests at different walking speeds. NAS = number of actual strides, NDS = number of detected strides, SE = stride-counting error, TD = total distance, ED = estimated distance, DE = distance error, WS = walking speed, WS = walking slowly, WQ = walking quickly, WN = walking at a normal speed.

						Proposed PDR Method		PDR with Weinberg Model		
Vol. No.	Trial No.	NAS	NDS	SE (%)	TD (m)	ED (m)	DE (%)	ED (m)	DE (%)	WS
1	1	70	69	1.4	94.4	98.2	4.0	104.5	10.2	WS
	2	68	68	0	94.4	99.1	5.0	104.2	10.3	WS
	3	66	66	0	94.4	97.2	2.9	103.7	9.8	WN
	4	65	65	0	94.4	94.3	0.1	100.9	6.8	WN
	5	58	58	0	94.4	96.4	2.1	97.6	3.3	WQ
	6	56	56	0	94.4	91.97	2.5	93.4	1.0	WQ
2	1	73	71	2.7	94.4	92.5	2.0	105.1	11.3	WS
	2	70	69	1.4	94.4	98.1	3.9	108.2	14.6	WS
	3	65	65	0	94.4	96.3	2.0	101.7	7.7	WN
	4	64	64	0	94.4	97.5	3.2	101.1	7.1	WN
	5	53	53	0	94.4	93.2	1.2	90.2	4.4	WQ
	6	54	54	0	94.4	91.9	2.6	91.1	3.5	WQ
Mean				0.5 ± 0.9			2.6 ± 1.3		7.5 ± 3.9	

**Table 6:** Results of indoor positioning: SE = stride counting error, EE = end-to-end error, DE = distance error.

Volunteers No.	Trial No.	Stride Count			EE (%)	DE (%)
		Actual	Detected	SE (%)		
1	1	123	123	0	5.7	2.6
	2	124	125	0.8	3.1	3.1
	3	124	124	0	6.2	2.5
2	1	116	117	0.8	3.8	2.9
	2	121	121	0	6.0	4.4
	3	125	127	1.6	7.4	2.2
Mean	-	-	-	0.5 ± 0.7	5.4 ± 1.6	3.0 ± 0.8

**Table 7:** Results of outdoor positioning, SE = stride-counting error, EE = end-to-end error, DE = distance error.

Volunteers No.	Trail No.	Stride Count			EE (%)	DE (%)
		Actual	Detected	SE (%)		
1	1	303	301	0.6	0.4	3.5
	2	281	278	1.0	2.7	1.0
	3	285	283	0.7	0.6	0.87
Mean	-	-	-	0.8 ± 0.2	1.2 ± 1.3	1.8 ± 1.5

**Table 8:** The comparison of our findings with those of related works. EE = end-to-end error, DE = distance error.

Works	$d_{ref}$ (m)	EE (%)	DE (%)	Number of Participants
[24]	68	1.3	2.1	3
[13]	86.4	0.48	2.0	3
[17]	190	-	0.45	1
[32]	224.6	0.42	0.22	1
Ours	153.6	5.4	3.0	2
	400	1.2	1.8	

## Data Availability

Not applicable.
